# Numerical analysis of wide-field optical imaging with a sub-20 nm resolution based on a meta-sandwich structure

**DOI:** 10.1038/s41598-017-01521-w

**Published:** 2017-05-02

**Authors:** Shun Cao, Taisheng Wang, Jingzhong Yang, Bingliang Hu, Uriel Levy, Weixing Yu

**Affiliations:** 1 State Key Laboratory of Applied Optics, Changchun Institute of Optics, Fine Mechanics & Physics, Chinese Academy of Sciences, No. 3888, Dongnanhu Road, Changchun, Jilin P. R. China; 2 University of the Chinese Academy of Sciences, Beijing, 10039 P. R. China; 3 Key Laboratory of Spectral Imaging Technology, Xi’an Institute of Optics and Precision Mechanics, Chinese Academy of Sciences, No. 17, Xinxi Road, Xian, 710119 P. R. China; 4 Department of Applied Physics, The Benin School of Engineering and Computer Science, The Center for Nanoscience and Nanotechnology, The Hebrew University of Jerusalem, Jerusalem, 91904 Israel

## Abstract

Biological research requires wide-field optical imaging techniques with resolution down to the nanometer scale to study the biological process in a sub-cell or single molecular level. To meet this requirement, wide-field structured illumination method (WFSIM) has been extensively studied. The resolution of WFSIM is determined by the period of the optical interference pattern. However, in traditional WFSIM this period is diffraction limited so that pattern having periodicity smaller than 100 nm cannot be generated and as a result achieving an imaging resolution better than 50 nm is a great challenge. Here, we demonstrate a wide-field optical nanoimaging method based on a meta-sandwich structure (MSS) model. It is found that this structure can support standing wave surface plasmons interference pattern with a period of only 31 nm for 532 nm wavelength incident light. Furthermore, the potential application of the MSS for wide-field super-resolution imaging is discussed and the simulation results show an imaging resolution of sub-20 nm can be achieved. The demonstrated method paves a new route for the improvement of the wide field optical nanoimaging, which can be applied by biological researchers to study biological process conducted in cell membrane, such as mass transportation and others.

## Introduction

Surface plasmons (SPs), formed by collective oscillations of free electrons of the metal, are evanescent surface electromagnetic waves exists at the interface between a noble metal and a dielectric^1, 2^. Their unique properties such as strong localization and large in-plane momentum have been extensively exploited for applications in a variety of fields including biosensing, wave guiding, photolithography, subdiffraction limited imaging, enhanced light-matter interactions and so on^3–9^. Typically, SPs have a higher wave vector (*k*_*sp*_) than that of light excited in air (*k*_*0*_) by carefully selecting the permittivity of dielectric and metallic materials. Therefore, the effective wavelength of SPs can be less than 100 nm in the visible waveband, making SPs good candidates for imaging resolution improvements. Recently, the application of SPs in super-resolution has been extensively studied in configurations such as perfect lens, plasmonic superlens and hyperlens^10–14^. More recently, SPs were introduced in far-field microscopy, i.e. plasmonic structured illumination microscopy (PSIM), to improve imaging resolution even further^15–19^. In those works, SP interference pattern was used for illumination, i.e. structured light was used to realize a lateral resolution enhancement of a fluorescence image. The resolution of PSIM is determined by *λ*_*em*_/(2NA + 2NA_eff_)^15, 16^, in which NA_eff_ = *k*_*sp*_/*k*_*em*_ with *k*_*em*_ stands for the wave vector of the emitted light. Apparently, higher *k*_*sp*_ leads to higher resolution. Previous studies show that subwavelength metallic structures can be used to excite SPs with a higher frequencies^15, 20^. In terahertz and infrared domains, graphene with outstanding electric, thermal and optical properties was utilized to realize SP wavevector which is much larger than that of the incident waves for applications in transformation optics, nano-imaging and tunable metamaterials^21–25^. In 2014, G. Bartal *et al*. demonstrated a SP focal spot with a size of less than 80 nm, which can be used as a super resolution virtual probe for direct measurements^26^. Many studies show that a hyperbolic metamaterial, which is essentially an anisotropic metamaterial, is able to support a very high *k*_*sp*_^9, 27–31^. Recently, our group designed a gradient permittivity meta-structure (GPMS), which consists of alternating metallic and dielectric films with a gradient permittivity and obtained sub-45 nm resolution^32^.

In this work, we proposed a simple and tunable meta-sandwich structure (MSS) which can support even higher *k*_*sp*_. By incorporating this structure in PSIM, an even higher imaging resolution can be achieved. The so called MSS consists of four simple stack of films: Ag/Al_2_O_3_/Ag/H_2_O. This stack is very flexible and allows to tune the periodic plasmonic interference pattern by simply changing the material and thickness of the dielectrics. It is found that the minimum period of the plasmonic interference pattern which can be obtained is 31 nm for a 532 nm illumination wavelength. As a result, a resolution of 16 nm could be achieved in one dimension in PSIM by employing the proposed MSS structure as the illumination source in plasmonic based microscopy.

## Results and Discussion

### Structure description and simulation results

**Figure 1:**
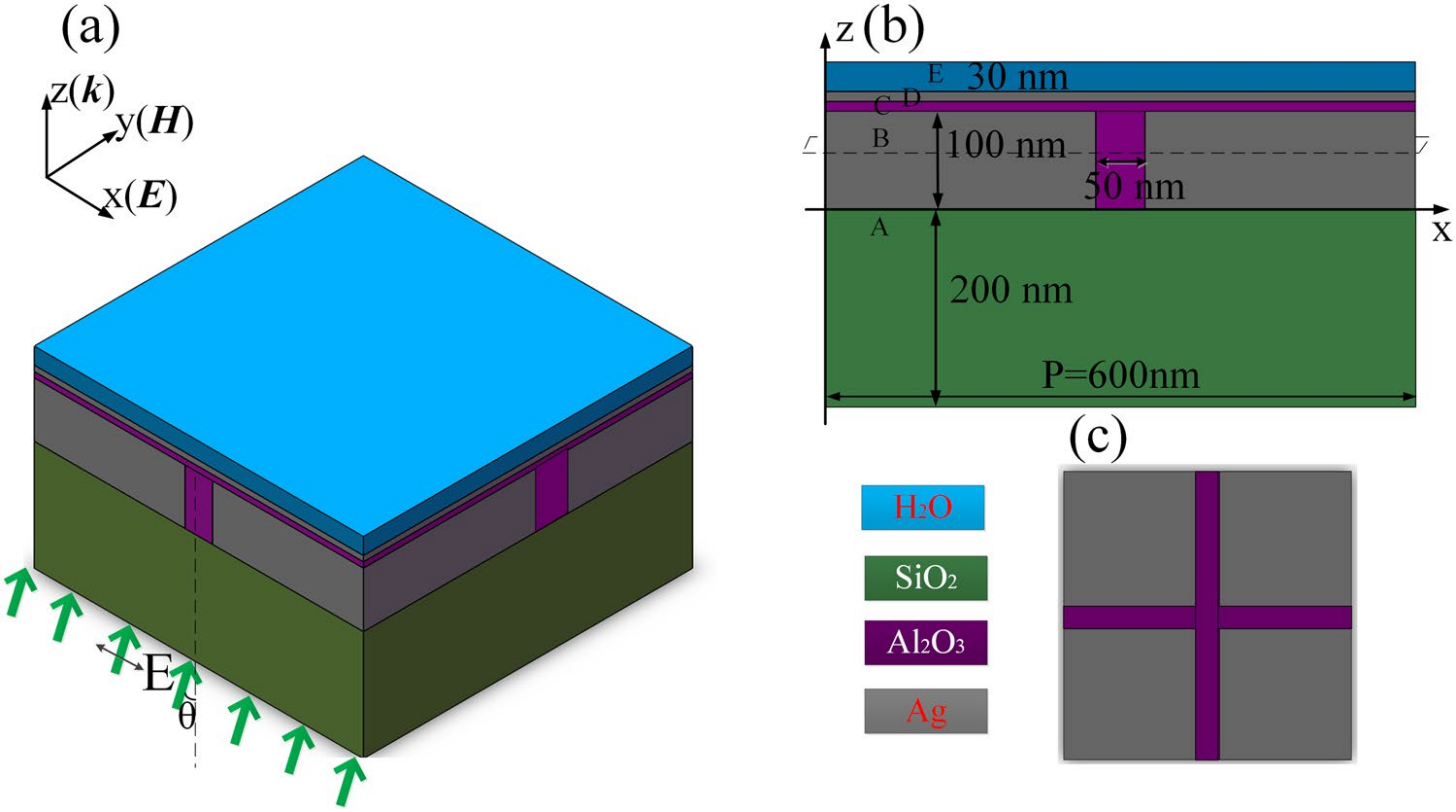
The schematic diagram of the meta-sandwich structure. (**a**) Perspective view and (**b**) cross sectional view, (**c**) top view of *Layer B*, marked as the dashed rectangle in (**b**).

The designed MSS for achieving a high SP wavevector of (*k*_sp_) in the optical domain, is shown in Fig. 1. Figure 1(a) and (b) show the perspective and the cross sectional views of the MSS, respectively. The parameters of the MSS geometry are shown in Fig. 1(b). The silver film in *Layer B* is 100 nm thick. There are periodic slits in the Ag film which are filled by dielectric material of Al_2_O_3_ (*ε*_*A*_ = 3.138). Figure 1(c) shows this feature of the layer. *Layers C* and *D* consist of Al_2_O_3_ and Ag with thickness of *d* = 10 nm each. *Layer E* is water, which is used to mimic aqueous environment for biological samples in structure illuminated microscopy. The unit cell is replicated as a periodic array, having period of 600 nm in both *x* and *y* directions.

**Figure 2:**
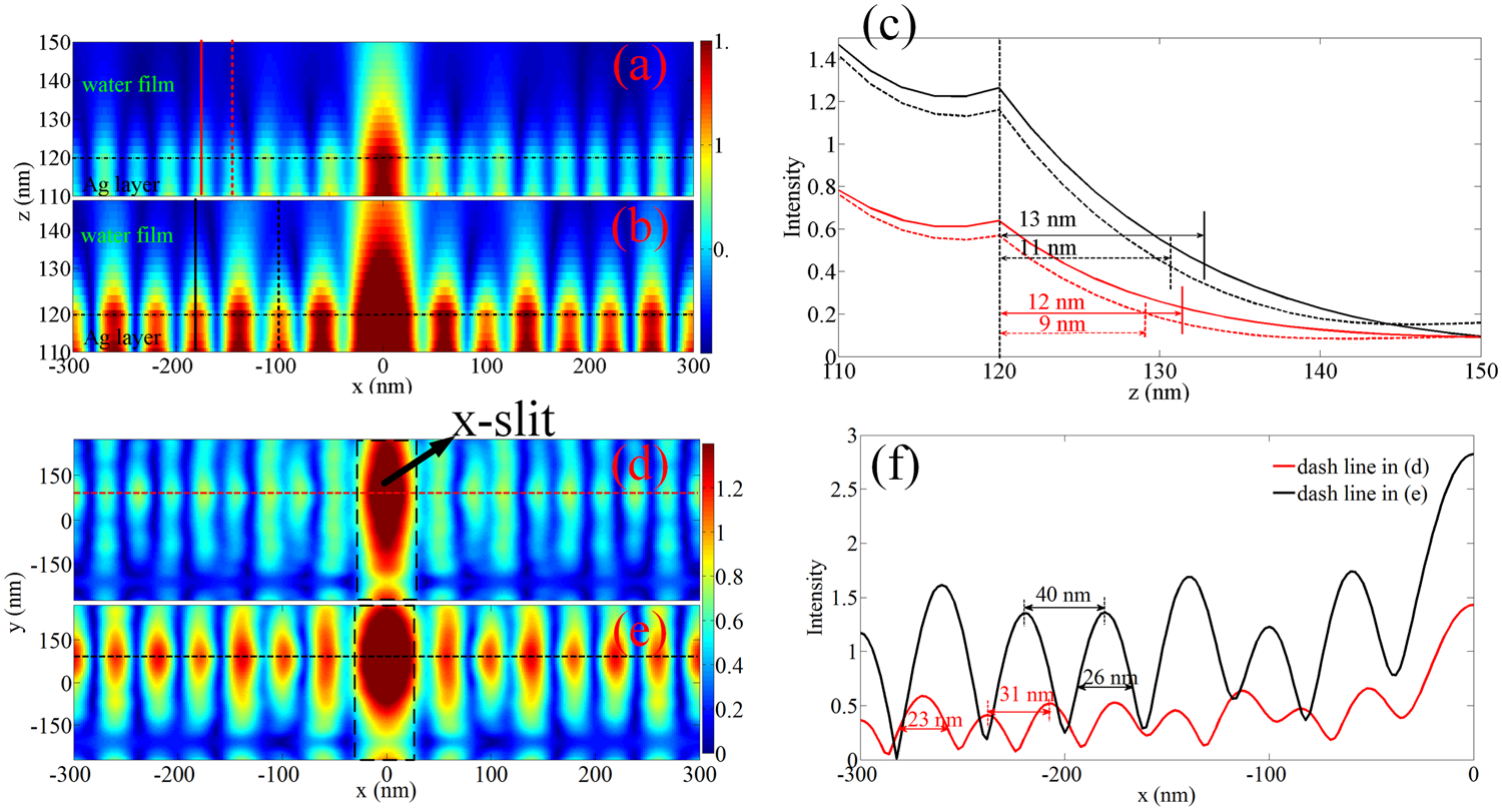
The distribution of the SW-SPs in the water film of the MSS. (**a**) and (**b**) The distribution of *x*-component of the electric field in *y* = 0 plane, when the dielectric materials in *Layer C* is Al_2_O_3_ and SiO_2_, respectively. (**c**) The intensity of the electric field along the vertical lines in (**a**) and (**b**): red for (**a**) and black for (**b**). (**d**) and (**e**) the distribution of *x*-component of the electric field in *z* = 122 nm plane, when the dielectric materials in *Layer C* is Al_2_O_3_ and SiO_2_, respectively. (**f**) The distribution of the electrical field intensity along the dashed lines, red for (**d**) and black for (**e**), respectively.

The distributions of the electric field in a single unit cell at different planes were calculated by the Frequency-domain field solver and the results are shown in Fig. 2. For comparison, another configuration with a SiO_2_ material in *Layer C* replacing the Al_2_O_3_ was also simulated. Figure 2(a) and (b) show the *x*-component of the electric field at plane *y* = 0 in *Layer D* (the uppermost silver layer) and *Layer E* (the water film) for *Layer C* with materials of Al_2_O_3_ and SiO_2_, respectively. The color bar in Fig. 2 denotes electric field intensity. It can be seen clearly that the standing wave (SW) pattern of SPs exists for both structures. However, the intensity of SW-SPs is not uniform in the water layer. In comparison with Fig. 2(b), the period of the SW-SPs in Fig. 2(a) is smaller. Figure 2(c) shows the electric intensity distribution along the lines (red lines in Fig. 2(a) and black lines in Fig. 2(b)) for both models. As can be clearly seen, the intensity of the SPs decays exponentially from *z* = 110 nm (the interface between *Layer C* and *Layer D*) to *z* = 120 nm (the interface between *Layer D* and *Layer E*). At *z* = 120 nm, the intensity of SP waves show a small peak which serve as an evident for the slight field enhancement at this point. This is because of the introduction of the second layer of Ag film which provides free electrons so that more SPs are excited and generated at the interface. For *z* > 120 nm, the intensity of the electric field decreases in exponential fashion again. The depth of penetration of the SPs into the water film is about 10 nm, as extracted from the curves in Fig. 2(c). The distribution of the *x*-component of the electric field for both models at plane *z* = 122 nm (2 nm above the interface of the Ag/water film) is shown in Fig. 2(d) and (e), respectively. Figure 2(f) is the electric field intensity distribution along the red dashed line in Fig. 2(d) and black dashed line in Fig. 2(e), respectively. Only half of the line is plotted due to the symmetry of the structure. It can be calculated from the red line that the period of the plasmonic interference pattern (the material of *Layer C* is Al_2_O_3_) is only 31 nm and the full width at half maximum (FWHM) is as small as 23 nm. However, when the material in *Layer C* is changed to SiO_2_, the period of the plasmonic interference pattern becomes larger, i.e. 40 nm, with a 26 nm FWHM (black line). Therefore, one can tune the period of the plasmonic interference pattern by simply changing the material in *Layer C*.

### The tunability of the period of plasmonic interference pattern in MSS

Based on the Equations of the analytical computation of the MSS model in the Supplementary Information, the wavevector of SPs in the MSS can be calculated and compared with the numerical results. For MSS filled with Al_2_O_3_ in *Layer C*, a wave vector of *β* = 0.0849 rad/nm for SPs can be calculated in the water layer, which corresponds to a plasmonic wavelength of 74 nm. Therefore, the period of the plasmonic interference pattern (half wavelength) is 37 nm, which is a bit larger than 31 nm obtained by the rigorous numerical simulation. When *Layer C* is filled with SiO_2_, the period of plasmonic interference pattern was calculated to be 46 nm, which is again a bit larger than 40 nm obtained by numerical simulation. However, these values are in good agreement in general. As a result, the validity of the MSS model is confirmed by both numerical and analytical methods. The physical mechanism of MSS is based on the symmetrical coupling of two short range surface plasmon polariton (SRSPP) modes in MSS as the model of MSS can be simplified to an Insulator-Metal-Insulator-Metal-Insulator (IMIMI) model. From Fig. 2(a) and (b), it can be found that electric field profiles are almost symmetric with respect to the center of the Ag slab. Therefore, this MSS can support SRSPP due to the mode overlap between modes on both sides of the center Ag slab. As the thickness of Al_2_O_3_ dielectric film is only 10 nm, two SRSPP waveguide modes will couple symmetrically in the Al_2_O_3_ dielectric film and therefore generate SPP with ultra-high wave-vector^33^. And wave-vector that the MSS supports can be calculated by the equations in Supplementary Information. The physical mechanism of MSS is different from our previous work^32^, where a multilayer gradient metamaterial film was employed to support the surface plasmon mode with a high wavevector and thus mode coupling and splitting effects do not exist.

**Figure 3:**
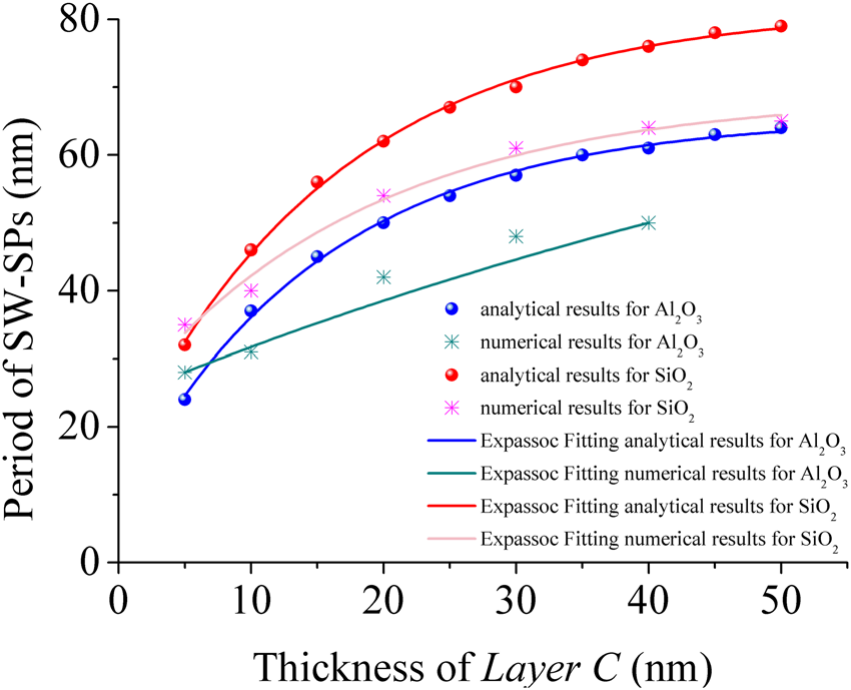
The period of plasmonic interference pattern as a function of the thickness of *Layer C* for different material combinations of Ag/Al_2_O_3_ and Ag/SiO_2_.

In order to show the tunability of the MSS model, the effect of different parameters including the thickness and material of *Layer C* on the period of SW-SPs were studied and the results are shown in Fig. 3. Circle and star points represent the results obtained by the analytical and numerical methods respectively. As shown in Fig. 3, in general the period of SW-SPs increases with the increase in thickness of *Layer C* and follows an ExpAssoc distribution form. We also note that the period is almost invariant as the thickness is larger than 40 nm. It should also be noted that the analytical results agree fairly well with the numerical ones for thin layers, but the deviation becomes larger with the increase in thickness. One possible explanation is that the analytical method for MSS model would be invalid when the thickness of *Layer C* is larger than 40 nm. Our model assumes that SPs can propagate through all the films and the SPs are evanescent waves. However, in practice SPs cannot not propagate into the upper films if the thickness of the film is larger than the penetration depth of SPs. When the thickness of Al_2_O_3_ is 40 nm, the calculated penetration depth of SPs is about 37 nm^1^, which is a bit shorter than the layer thickness. As a result, the analytical result becomes inaccurate. Similar issue occurs for the case of SiO_2_.

When comparing the blue and the red line in Fig. 3, it can also be found that the period of SW-SPs decreases if the permittivity of the material in *Layer C* reduces. Here, due to its small propagation loss, Ag is chosen as the metallic layer and hence SPs with a large propagation length can be supported. Many efforts were devoted to extend the propagation length of SPs such as directional surface plasmonic excitation, although they are working in a limited space^34, 35^. Therefore, one can use dielectric materials with a higher permittivity as *Layer C* and at the same time use metals with both small real and imaginary parts of the permittivity to further increase the wavevector of SPs. By mixing or doping dielectrics or semiconductors in metallic films such as Ag one can realize such effective metals with the required permittivitiy^36^.

In our MSS, metallic slit array was used to generate SW-SPs. Other coupling schemes such as half circle slits^26^ or special designed metadevices^37^ could also be used to generate additional forms of SPs interference patterns. From the color bar in Fig. 2(a) and (b), the intensity of the SW-SPs in the water film is about 0.6 *I*_*0*_ (*I*_*0*_ is the intensity of incident light), which is much stronger than that obtained by traditional multilayer metamaterials^27^. This will benefit the application of biological samples imaging. However, the penetration depth of the SW-SPs in the water medium is only about 10 nm, which is much smaller than that in the conventional Ag-air structure.

### Effect of the fabrication errors on the period of plasmonic interference pattern in MSS

Fabrication errors cannot be avoided so that the MSS structure cannot be perfectly fabricated in reality. To investigate the effect of fabrication errors on the period of plasmonic standing wave pattern in MSS, four error sources are considered, including surface roughness of the upper silver and Al_2_O_3_ layers, slit width, and the thickness of upper and bottom Ag films.

**Figure 4:**
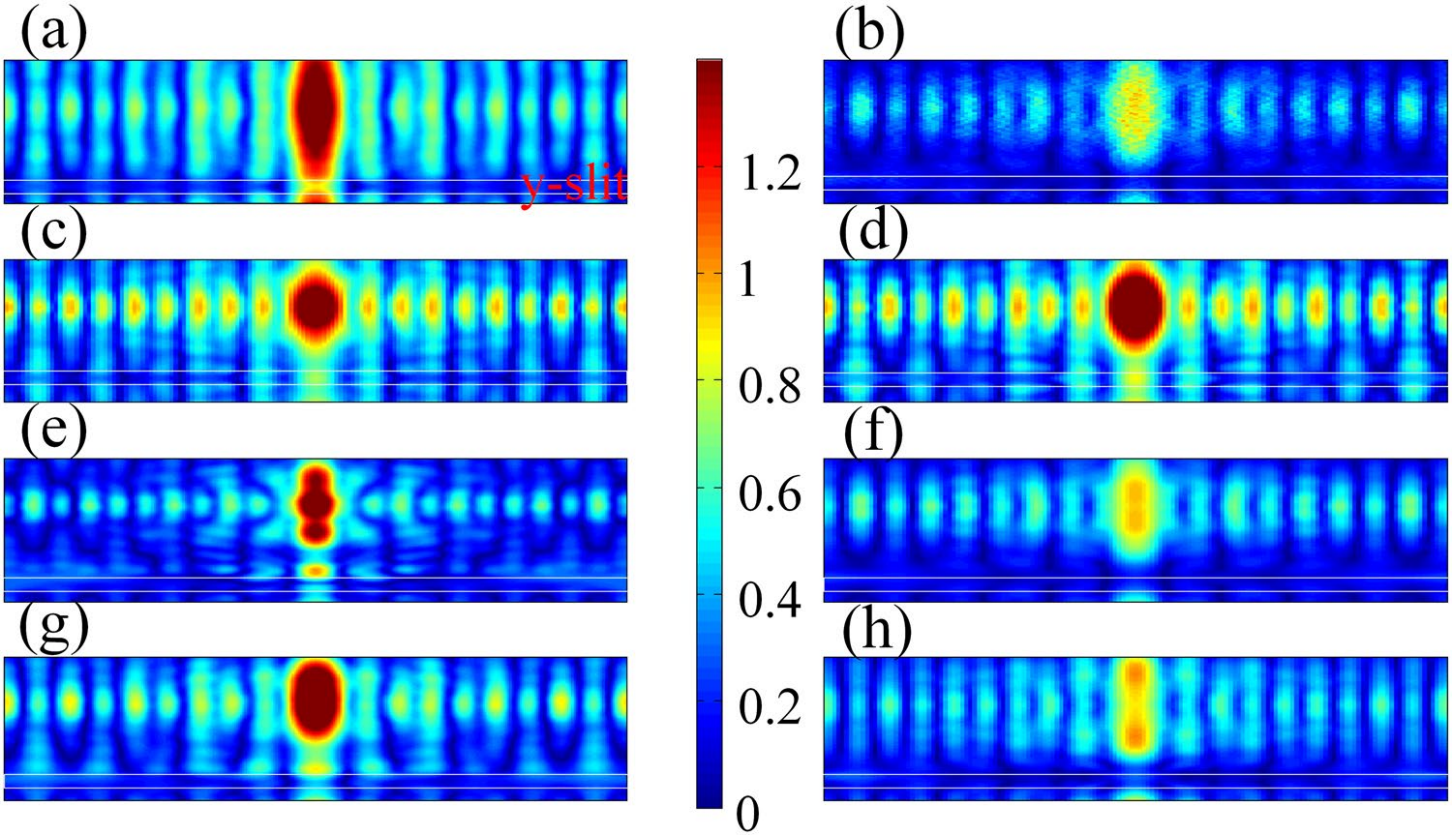
The distribution of the SW-SPs in the water film of MSS under different parameters errors: (**a**) no fabrication error, (**b**) the surface roughness of up Ag and Al_2_O_3_ films, the width of slits: (**c**) 45 nm (with 5 nm width error) and (**d**) 55 nm (with 5 nm width error), the thickness of up Ag layer: (**e**) 8 nm (with 2 nm thickness error) and (**f**) 12 nm (with 2 nm thickness error), and the thickness of bottom Ag layer: (**e**) 98 nm (with 2 nm thickness error) and (**f**) 102 nm (with 2 nm thickness error).

Figure 4(a) shows the distribution of the *x*-component of the electric field for MSS at plane 2 nm above the Ag/water interface under ideal conditions. For surface roughness error, we set the surface roughness (P-V value) of the upper silver and Al_2_O_3_ films to be less than 0.2 nm. After numerical calculation, the distribution of the *x*-component of electric field at plane *z* = 122 nm is shown in Fig. 4(b). From this figure, one can clearly find that the SW-SPs still exist. The period of SW-SPs is almost identical in comparison with that shown in Fig. 4(a). However, the maximum intensity of the electric field in water film is about 0.4 *I*_*0*_, which is weaker than that obtained for the ideal conditions. Figure 4(c) and (d) show the distribution of the *x*-component of the electric field at plane *z* = 122 nm when a 5 nm of slit width error is applied, that is to say, the width of the slit is 45 nm or 55 nm, respectively. From these figures, one can clearly find that the electric field distribution of SW-SPs barely changes. In fact, the state of the art of electron beam lithography is able to control the line width within 5 nm. Therefore, by carefully controlling the fabrication process, the fabrication tolerance on slit width could have no effect on the period of plasmonic interference pattern in the MSS. For the film thickness of the upper silver film, an error of 2 nm is applied. Figure 4(e) and (f) show the electric field distribution for Ag film with a thickness of 8 and 12 nm respectively. As can be seen, the period of SW-SPs was 26 nm in Fig. 4(e), which is smaller than 31 nm in Fig. 4(a). On the contrary, the period of SW-SPs in Fig. 4(f) is 36 nm, which is larger. As a result, the error of the thickness of upper Ag layer has a significant influence on the period of plasmonic interference pattern in MSS and hence on the final imaging resolution. Therefore, the thickness of the upper silver film needs to be controlled carefully. However, for bottom silver film the error on the thickness has no effect on the period of SW-SPs as shown in Fig. 4(h).

### Super-resolution imaging performance of the MSS

**Figure 5:**
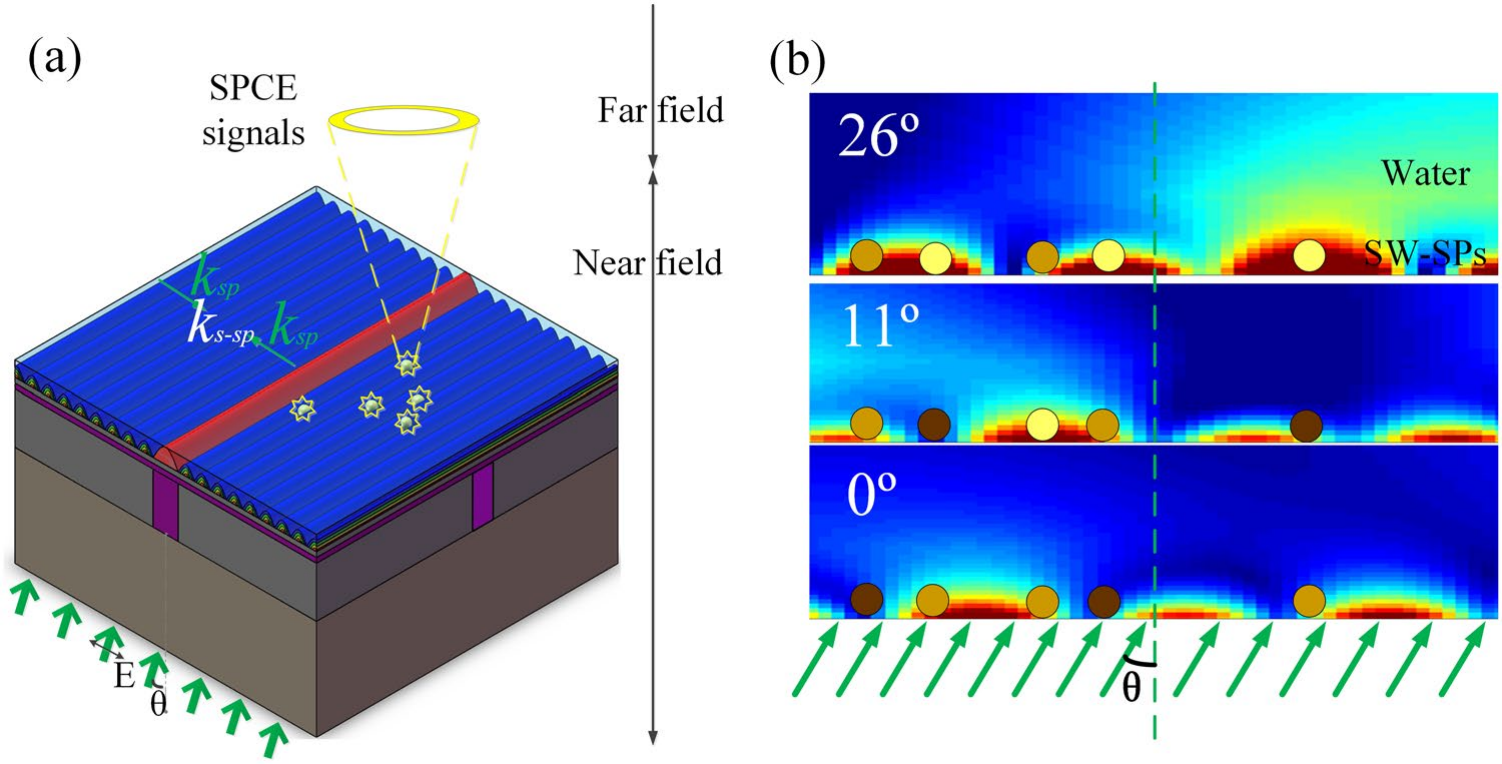
The schematic diagram of the MSS used in PSIM. (**a**) Optical configuration of SPs generated by MSS. The plasmonic interference pattern is generated by two adjacent counter propagating SPs and used to excite the quantum dots (or fluorescent beads) in water film, and (**b**) schematic demonstration of 120° phase shift of the SPs interference pattern with different incident angles (0°, 11°, and 26°).

The proposed MSS can be applied in super-resolution imaging with a plasmonic structure illumination microscopic mode. Figure 5 shows how the proposed MSS is used for SIM imaging. To demonstrate its capability in resolution improvement, the image of a single point particle (quantum dot (QD)) was used as the object. The QD object was placed in water on the top surface of the Ag film. As can be seen in Fig. 5(a), the plasmonic interference pattern is used as the structured light to illuminate the QD object and the surface plasmon-coupled emission (SPCE) signal of the QD is recorded in the far field. The phase shift of SPs can be obtained by changing the incident angle *θ* of the incident light as is shown in Fig. 5(b) ^15, 16^. This relationship between the SPs phase shift and the incident angle was clearly shown in the simulation results in Fig. 5(b). The QDs is illuminated with SPs with different phase shifts (see Fig. 5(b)), i.e. a sequence of three phase shifts of 0°, 120°, −120°. In order to generate a reconstructed image, the same method as in our previous work is applied. More details can be found elsewhere^32^. The numerical algorithm used to reconstruct the high-resolution image is the same as that used in standing-wave total internal reflection fluorescence^38^.

**Figure 6:**
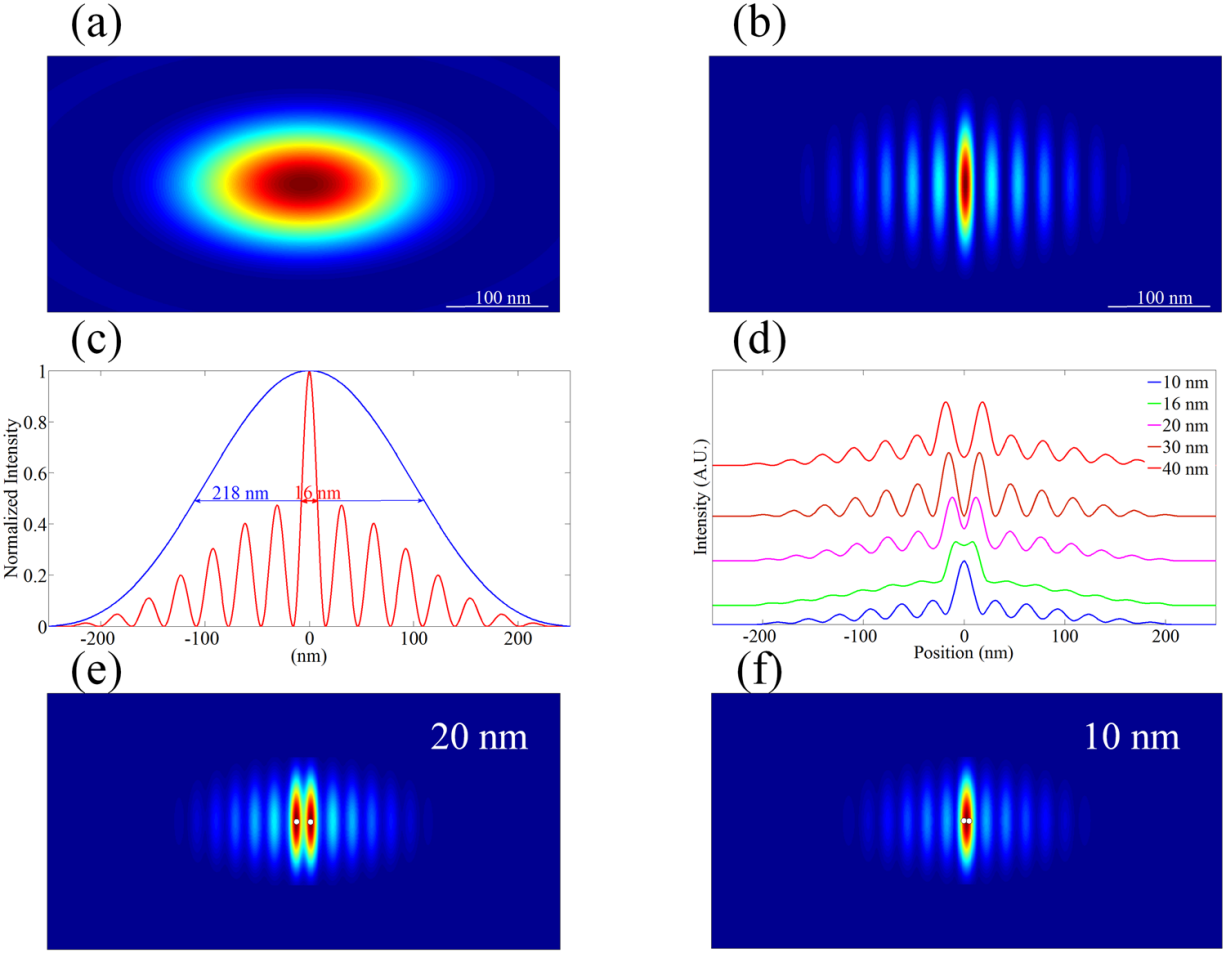
The simulation results of the MSS imaging performance. Point spread function of (**a**) a diffraction-limited system, (**b**) reconstructed image in *x*-direction, (**c**) FWHM comparison between conventional epi-fluorescence microscopic image (blue curve) and the super-resolution image by using the MSS (red line) in PSIM. (**d**) Illustration of resolving capability of MSS-PSIM of two PEs with size of 10 nm and separated with different distances of 10, 16, 20, 30 and 40 nm in *x*-direction. (**e**) The reconstructed image of two PEs separated by 20 nm. (**f**) The reconstructed image of two PEs separated by 10 nm. The white circles denote the positions and distributions of PEs.

In our numerical model, a 10 nm point emitter (PE) model with emission wavelength of 600 nm was used to simulate the point spread function (PSF) of the standard system and characterize the resolution of the MSS based PSIM. Intensity distribution of the PE is step-function. The fluorescent signal is detected by an immersion oil objective with a NA of 1.42 in the simulated model. The results are shown in Fig. 6. Figure 6(a) shows the image of the PSF of the PE obtained by a conventional homogeneous illuminating method. Figure 6(b) shows the reconstructed image of the PE obtained by PSIM for an incident light with a wavelength of 532 nm. As can be seen clearly in Fig. 6(b), there exist sidelobe artifacts surrounding the central spot, which is similar to other super-resolution methods based on the interference method^39, 40^. These artifacts can be eliminated by using appropriate post-processing method so that the image quality can be further improved^41^. Figure 6(c) shows a comparison of the PSF profile across the *x*-axis of Fig. 6(a) and (b). As can be seen, the FWHM of the conventional epi-fluorescence microscopic image is about 218 nm. In contrast, the FWHM is only about 16 nm for the image obtained by PSIM with a MSS structure. This means that the imaging resolution has been improved 13.6 fold by introducing the MSS structure in PSIM. This result is better than SIM^42^ and previous reported PSIM^15, 16, 32, 43^. It should be noted that two-dimensional enhancement in imaging resolution of the PE can also be obtained by using the SPs pattern generated in MSS to illuminate the PE in both *x* and *y* directions. This can be achieved by using an incident light wave with *y*-polarized state instead. Moreover, the resolving capability of MSS-PSIM can be obtained by imaging two-point emitter objects with different separation distance as shown in Fig. 6(d). Again, the processing details can be found elsewhere^38^. These cross sectional profiles show the imaging enhancement in *x*-direction by using a one-dimensional SPs interference pattern. Based on the Rayleigh criterion, it is shown that MSS-PSIM has the capability to resolve two objects with a size of 10 nm and separated by about 16 nm. Figure 6(e) and (f) show the images of two PEs with 20 nm and 10 nm separations, respectively. We can see from these two figures, MSS-PSIM has the ability to resolve two PEs separated by about 20 nm but can not resolve two PEs with 10 nm separations.

## Conclusions

In this work, a meta-sandwich structure (MSS) was demonstrated theoretically and numerically. The MSS consists of only four layers of alternating dielectric/metallic films. In comparison with traditional multilayer metamaterial structure and gradient permittivity meta-structure, MSS is more simple and elegant and it supports SPs wave with even higher wavevectors. The validity of the MSS in supporting short wavelength SPs wave was proved by employing both rigorous numerical and analytical methods. Good agreement is obtained between the two approaches. In addition, the period of the plasmonic interference pattern can be tuned by changing the parameters of the MSS. It is found that the period of the SPs interference pattern is only about 1/17 of the wavelength of the incident light. The potential application of this deep subwavelength SPs interference pattern for super-resolution imaging was discussed. It was shown that the reconstructed quantum dot image provides a 13.6-fold improvement in resolution in comparison with that of the conventional epifluorescence microscopy. All of these advantages of MSS point towards potential applications in the field of super-resolution biomedical imaging as well as in nanolithography.

## Methods

All the numerical simulations, including modeling and analysis of the MSS were done with commercial finite-difference time domain (FDTD)^44^ software package (Lumerical FDTD Solutions). The three-dimensional (3D) simulations were performed with a TM polarization (*x*-polarized) plane wave source at *λ*_0_ = 532 nm incident in the z direction. This polarized incident light is used to generate *x*-dimensional SPs. The *y*-dimensional SPs can be achieved by using an incident light wave with a *y*-polarized state instead. The incident angle is set as *θ*_0_ = 0, i.e. normal incidence. Periodical boundary conditions (PBC) in both *x* and *y* directions and perfectly matched layers (PML)^45^ as absorbing boundary conditions in *z* direction were used. The permittivity of Ag was assumed to be −11.75 + 0.37i at *λ*_0_ = 532 nm^46^. The FDTD region was set as 0.6 × 0.6 × 2 μm^3^. In the regions where SPs exist, finer meshes with smaller cubes of 2 × 2 × 2 nm^3^ was applied while a coarse meshing was constructed elsewhere. Frequency-domain field and power monitors were used to investigate the distributions of the electric field at *y* = 0 nm and *z* = 122 nm in the simulations.

## Electronic supplementary material

Supplementary Info
