# Supplementary material for: Numerical analysis of wide-field optical imaging with a sub-20 nm resolution based on a meta-sandwich structure
Source: Sci Rep. 2017 May 2;7:1328. doi: 10.1038/s41598-017-01521-w (PMC5431004; doi:10.1038/s41598-017-01521-w)
Supplement: Supplementary file 1 — Supplementary Info [file 41598_2017_1521_MOESM1_ESM.doc]

**Numerical analysis of wide-field optical imaging with a sub-20nm resolution based on a meta-sandwich structure**

**Shun Cao,^1, 2^ Taisheng Wang,^1^ Jingzhong Yang,^1, 2^ Bingliang Hu,^3^ Uriel Levy,^4^**

**and Weixing Yu^3, *^**

^1^State Key Laboratory of Applied Optics, Changchun Institute of Optics, Fine Mechanics & Physics, Chinese Academy of Sciences, No.3888, Dongnanhu Road, Changchun, Jilin, P. R. China

^2^University of the Chinese Academy of Sciences, Beijing, 10039, P. R. China

^3^Key Laboratory of Spectral Imaging Technology, Xi’an Institute of Optics and Precision Mechanics, Chinese Academy of Sciences, No.17, Xinxi Road, Xian 710119, P. R. China

^4^Department of Applied Physics, The Benin School of Engineering and Computer Science, The Center for Nanoscience and Nanotechnology, The Hebrew University of Jerusalem,

Jerusalem 91904, Israel

Correspondence should be addressed to W.Yu. (Email: yuwx@opt.ac.cn)

**Analytical computation of the MSS model**

For the sake of further understanding the physical mechanism of the MSS, the wavevector of SPs supported by it is derived analytically by solving the Maxwell equations. Supplementary Figure 1 shows the analytical computing model of MSS and the permittivity of the material in each film is also shown in the figure. The coordinate system of *x-z* axis of the analytical model is different from that of the MSS in Fig. 1 for the convenience of theoretical analysis. A TM polarized electromagnetic wave incidents along *z* axis. It is hypothesized that the SPs is already excited at the interface between *Layer A* and *Layer B*.

From the Maxwell equations, one can get the components of the electric and magnetic fields in MSS. The similar analytical equations can be found in Reference 31.

Finally, one can get the following equations,

$A=\frac{k_{2z}/\varepsilon_{2}+k_{1z}/\varepsilon_{1}}{k_{2z}/\varepsilon{}_{2}-k_{1z}/\varepsilon_{1}}$ (1)

$B=e^{-2k_{3z}(d_{2}+d_{3})}\frac{-(k_{4z}/\varepsilon_{4}-k_{3z}/\varepsilon_{3})}{k_{4z}/\varepsilon_{4}+k_{3z}/\varepsilon_{3}}$ (2)

$Ae^{2k_{2z}d_{2}}=\frac{B*(k_{2z}/\varepsilon{}_{2}+k_{3z}/\varepsilon_{3})e^{2k_{3z}d_{2}}+k_{2z}/\varepsilon_{2}-k_{3z}/\varepsilon_{3}}{B*(k_{2z}/\varepsilon{}_{2}-k_{3z}/\varepsilon_{3})e^{2k_{3z}d_{2}}+k_{2z}/\varepsilon{}_{2}+k_{3z}/\varepsilon_{3}}$ (3)

In above equations,

$k_{iz}=\sqrt{\beta^{2}-k_{0}^{2}\varepsilon_{i}}$ (*i*=1-4) (4)

Where *k_iz_* and *β* stand for the component of the wave vector of SPs in the *ith* layer that is perpendicular and parallel to the interface in MSS respectively, *k_0_* represents the wave vector of incident light and *d_i_* (*i*=1-4) stands for the thickness of the *ith* layer.


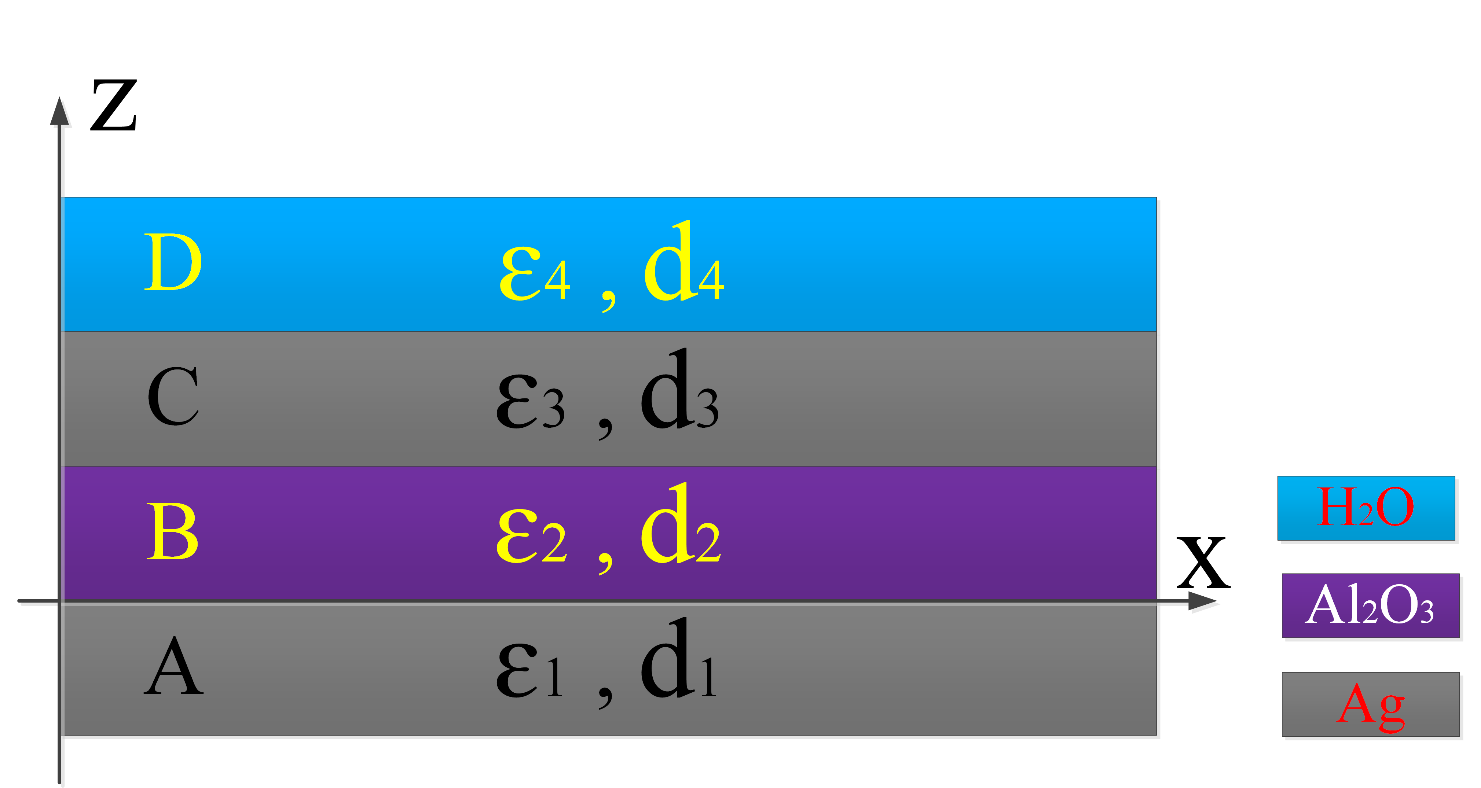


**Supplementary Figure 1. The schematic diagram of the model of MSS used in analytic method.** *ε_i_* and *d_i_* stand for the permittivity and thickness of the material in each layer.
